# Protein kinase function of pyruvate kinase M2 and cancer

**DOI:** 10.1186/s12935-020-01612-1

**Published:** 2020-10-29

**Authors:** Xun Chen, Shangwu Chen, Dongsheng Yu

**Affiliations:** 1 Department of Oral and Maxillofacial Surgery, Guangdong Provincial Key Laboratory of Stomatology, Guanghua School of Stomatology, Sun Yat-sen University, 56 Lingyuan West Road, Guangzhou, 510055 People’s Republic of China; 2 Department of Biochemistry, Guangdong Key Laboratory of Pharmaceutical Functional Genes, MOE Key Laboratory of Gene Function and Regulation, State Key Laboratory for Biocontrol, School of Life Sciences, Sun Yat-sen University, Guangzhou, 510275 People’s Republic of China

**Keywords:** Pyruvate kinase M2, Protein kinase, Glycolytic pathway, Non-metabolic function, Tumorigenesis

## Abstract

Pyruvate kinase is a terminal enzyme in the glycolytic pathway, where it catalyzes the conversion of phosphoenolpyruvate to pyruvate and production of ATP via substrate level phosphorylation. PKM2 is one of four isoforms of pyruvate kinase and is widely expressed in many types of tumors and associated with tumorigenesis. In addition to pyruvate kinase activity involving the metabolic pathway, increasing evidence demonstrates that PKM2 exerts a non-metabolic function in cancers. PKM2 has been shown to be translocated into nucleus, where it serves as a protein kinase to phosphorylate various protein targets and contribute to multiple physiopathological processes. We discuss the nuclear localization of PKM2, its protein kinase function and association with cancers, and regulation of PKM2 activity.

## Background

Glycolysis is an important process of glucose degradation in which a glucose is broken down into two pyruvates. The basic metabolic function of pyruvate kinase (PK) in the glycolytic pathway is to catalyze the final step, in which a phosphate group in phosphoenolpyruvate (PEP) is transferred to ADP, yielding pyruvate and ATP [1]. Pyruvate kinase has four different tissue-specific isozymes in animals, PKL, PKR, PKM1, and PKM2. The L and R isozymes are expressed in the liver (L) and red blood cells (R), whereas PKM2 is expressed in early embryonic cells and other proliferating cells, and PKM1 is expressed in the brain, skeletal muscle, and heart which need high energy [2]. Pyruvate kinase L and R are encoded by the PKLR gene, and PKM1 and PKM2 are transcribed from the PKM gene via alternative splicing. PKM2 possesses the PKM2-specific exon 10 and lacks the PKM1-specific exon 9. The splicing process is regulated by splicing factors of the heterogeneous nuclear ribonucleoprotein A1 (hnRNPA1) and A2 (hnRNPA2) and polypyrimidine tract binding protein (PTB) [3]. The expression of these splicing factors is induced by transcription factor c-Myc and correlated with PKM2 level in tumors [3].

Pyruvate kinase can be present as a tetrameric or a dimeric form. The tetrameric structure is an active form with high binding affinity to PEP, while the dimeric form is less active with low binding affinity to PEP. The single exon difference between PKM1 and PKM2 leads to important function distinctions. PKM1 constitutively oligomerizes to a tetramer under physiological conditions, while PKM2 may be present as dimer or tetramer depending on the corresponding regulators. PKM1 and PKM2 are subject to differential allosteric regulation and covalent modification. Fructose-1,6-bisphosphate (FBP), a glycolytic intermediate, preferentially binds to PKM2, but not PKM1, and consequently increases the affinity of PKM2 to PEP [4]. In addition to FBP, many other metabolites [5], amino acids [6], and small molecules [7–9] are involved in the regulation of PKM2 activity. The binding of small molecule PKM2 activators to PKM2 promotes tetramer formation, constitutively activating PKM2 and suppressing tumorigenesis [8]. Post-translational modification of PKM2 such as through phosphorylation, acetylation, or oxidation facilitates the low activity of dimeric PKM2 [10]. Thus, pyruvate kinase activity can be regulated by altering its conformation. Constitutive activity of a tetramer such as PKM1 allows it to serve as pyruvate kinase in cytosol, favoring the glycolytic process and energy generation, while the less active dimeric PKM2 promotes the accumulation of glycolytic intermediates and subsequent biosynthesis in tumor cells. Importantly, the PKM2 dimer can be imported to the nucleus and function as a protein kinase [11].

Evidence supports a potential role of PKM2 in tumorigenesis. As an embryonic isoform, PKM2 is reactivated in tumors and overexpressed in multiple cancer types [12–15]. The alteration in PKM2 activity is related to cellular proliferation and tumor growth [8, 15, 16]. The deletion of PKM2 in normal cells results in the expression of PKM1 and induces proliferation arrest by impairing nucleotide production and subsequent DNA synthesis [2]. A switch from PKM1 to PKM2 has been detected in various cancers, and a reverse isoform switch from PKM2 to PKM1 has been found to inhibit aerobic glycolysis and reduce tumorigenesis in a nude mouse xenograft model [13, 14]. The oncogenic transcription factor c-Myc induces transcription of splicing factors to ensure PKM2 expression required for cancer cell proliferation and metabolism [3]. Protein kinase Bβ (AKT2) induces expression of PKM2 and PKM2-mediated STAT3 up-regulation and NF-κB activation facilitates invasion of cancer cells and tumor metastasis in nude mice [17]. PKM2 also induces tumor angiogenesis through activation of NF-κB and HIF-1α [18].

## Non-metabolic function of PKM2

**Fig. 1:**
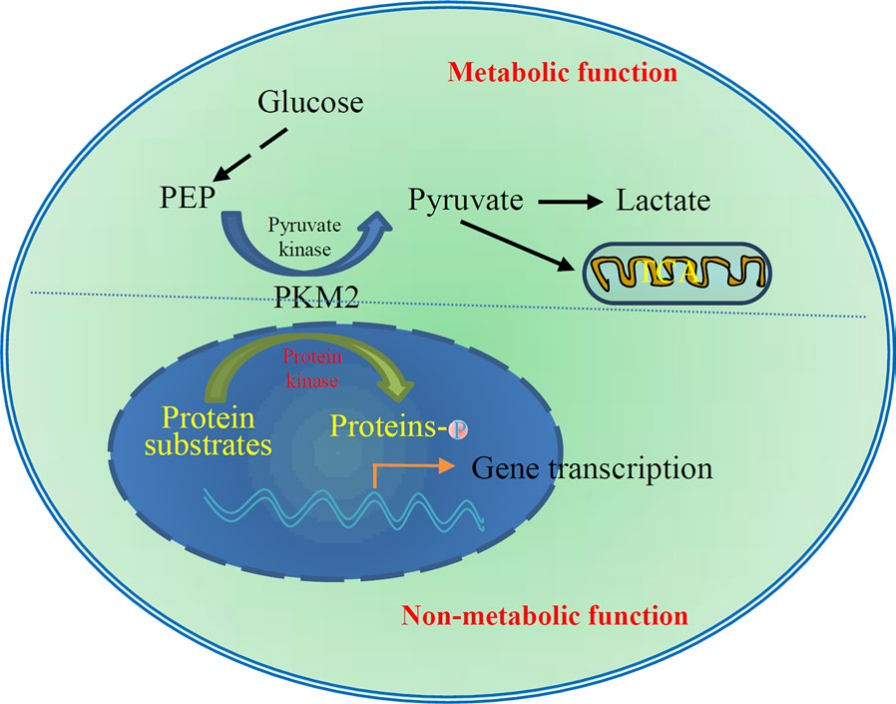
Metabolic and non-metabolic nuclear function of PKM2. PKM2 was originally known to serve as a pyruvate kinase in the glycolytic pathway, where it transferred a phosphate group from phosphoenolpyruvate to ADP, producing pyruvate and ATP. In addition to this so-called metabolic function, increasing evidence demonstrates that PKM2 can function as a protein kinase to phosphorylate a variety of protein targets and be involved in multiple physiopathological processes

Independent of its pyruvate kinase activity, PKM2 exerts non-metabolic functions such as protein kinase activity and transcriptional co-activation (Fig. 1). PKM2 has been observed to transfer to the nucleus, phosphorylate protein histone, and activate gene transcription, acting non-metabolic functions in cancer cells [18–21]. For example, epidermal growth factor receptor (EGFR) signaling promotes transfer of PKM2 into the nucleus, and the nuclear PKM2 binds to phosphorylated-Tyr333 of β-catenin [19]. The complex of PKM2/β-catenin is recruited to the promoter of CCND1, which encodes for cyclin D1, leading to cyclin D1 expression and consequently promoting tumor development and tumor cell proliferation [19]. Evidence supports a non-metabolic function of PKM2 in EGFR-induced tumorigenesis [19, 22].

**Fig. 2:**
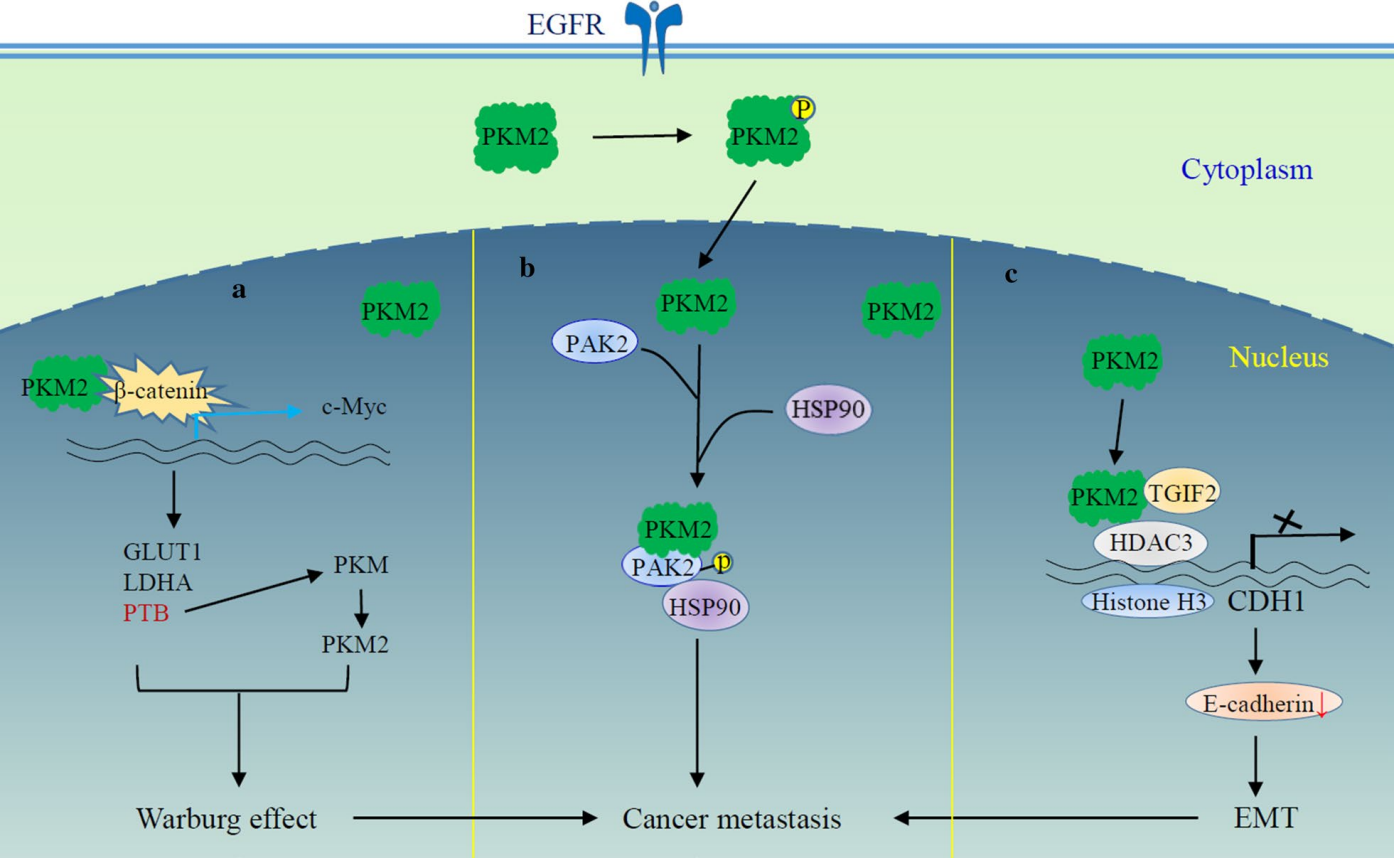
A schematic diagram illustrating the regulation of PKM2 on the Warburg effect, cancer metastasis and EMT. **a** Proposed mechanism of PKM2-regulated the Warburg effect. EGFR engagement facilitates PKM2 nuclear translocation. Nuclear PKM2 serves as a coactivator of β-catenin to activate expression of c-Myc, leading to the up-regulation of GLUT1, LDHA and PTB-dependent PKM2 expression. These glycolytic enzymes promote the Warburg effect. **b** PKM2 promotes cancer invasion and metastasis. Nuclear PKM2 interacts directly with and phosphorylates PAK2, a serine–threonine kinase with a major role in regulating cell mobility. The phosphorylation stabilizes PAK2 by facilitating HSP90 association to PAK2 and thus prevents ubiquitination and proteasomal degradation of PAK2, which increase the cell invasion ability and promote tumor metastasis. **c** PKM2 regulates EMT. Nuclear PKM2 dimer binds to TGIF2 and promotes its degradation through ubiquitin-proteasome system. This facilitates the recruitment of HDAC3 to the CDH1 promoter, which subsequently deacetylates histone H3 and inhibits CDH1 transcription. Down-regulation of E-cadherin expression induces EMT and promotes tumor cell invasion and metastasis CDH1, E-cadherin; EMT, epithelial mesenchymal transition; GLUT1: glucose transporter 1; HDAC3: histone deacetylase 3; HSP90: heat shock protein 90; LDHA: lactate dehydrogenase A; PAK2: p21 protein activated kinase 2; PTB: polypyrimidine tract binding protein; TGIF2: TGF-β-induced factor homeobox 2

The non-metabolic functions of PKM2 contribute to multiple processes of tumor pathology. In addition to tumor development, PKM2 is involved in regulation of the Warburg effect (Fig. 2) [23], cancer metastasis [24], epithelial mesenchymal transition (EMT) [25–27], gene expression [20], mitosis [28, 29], cellular proliferation [30, 31], apoptosis [32], DNA damage response [33], and exosome secretion [34] (see discussion below). PKM2 also interacts with some tyrosine kinases, acting as a signaling regulator in the cytoplasm. Cytosolic PKM2 interacts with mutant EGFR and HSP90, and consequently stabilizes EGFR [35]. This may contribute to EGFR-dependent tumorigenesis and drug resistance to EGFR tyrosine kinase inhibitor. Interaction of Src kinase and PKM2 is potentially associated to the metastasis of liver cancer [36]. In addition, PKM2 may serve as an epigenetic modulator by blocking nucleosome repositioning in chromatin via blockade of the Chromodomain Helicase DNA binding protein-7 mediated sliding of nucleosome [37].

## Roles of PKM2 in cancer

PKM2 is involved in many pathological processes of numerous tumor types such as gastrointestinal cancer, hepatocellular carcinoma and lung cancer. PKM2 can promote tumor growth, metastasis and chemo-resistance by directly regulating tumor cell metabolism as an enzyme or regulating different signaling pathways as a nuclear transcription cofactor [38, 39]. For example, PKM2 promotes tumor growth and suppresses cell apoptosis through regulating Bcl-xL transcription in gastric cancer cells [40] or regulating NF-κB/p65 and HIF-1α activation [18] and mitogen-activated protein kinases (MAPKs) [41] in pancreatic tumor cells. PKM2-mediated EMT is critical for colon cancer cells to acquire the invasive potential. EMT stimulation induces direct interaction of nuclear PKM2 with TGF-β-induced factor homeobox 2 (TGIF2), which recruits histone deacetylase 3 to the E-cadherin promoter, leading to deacetylation of histone H3 and suppression of E-cadherin transcription [26]. PKM2 also enhances tumor cell chemo-resistance [42–44]. Increase of PKM2 production by alternative splicing promotes gemcitabine resistance in pancreatic cancer cells [45]. PKM2 modulates the sensitivity of colorectal cancer cells to gefitinib through up-regulation of STAT3 activation [42]. miR-122 targets PKM2 in the colon cancer cells, and forced expression of miR-122 re-sensitizes 5-fluorouracil (5-FU)-resistant cells to 5-FU by inhibiting PKM2 [43]. Silencing PKM2 and kidney-type glutaminase expression significantly reverses the resistance of colorectal cancer cells to oxaliplatin [46]. Inhibition of PKM2 in esophageal squamous cell carcinoma cells significantly decreases cisplatin resistance and increases apoptosis by inactivating the pentose phosphate pathway [47].

High PKM2 expression is significantly associated with reduced overall survival in hepatocellular carcinoma [48]. PKM2 promotes metastasis of hepatocellular carcinoma by recruiting myeloid-derived suppressor cells [49]. Circular RNA MAT2B promotes hepatocellular carcinoma progression by enhanced glycolysis through activating the circMAT2B/miR-338-3p/PKM2 axis under hypoxia [50]. PKM2 nuclear translocation and activation is required for the aerobic glycolysis-driven hepatocarcinogenesis [51]. The m6A demethylase-mediated demethylation of PKM2 mRNA promotes hepatocellular carcinoma tumorigenesis [52]. In addition, knockdown of PKM2 in lung cancer suppresses tumor growth and invasion [53] and enhances the efficacy of docetaxel [54] and radiosensitivity [55]. Pharmacologic activation of PKM2 by small molecules reduces lung tumor xenograft growth [56].

## Nuclear localization of PKM2

**Fig. 3:**
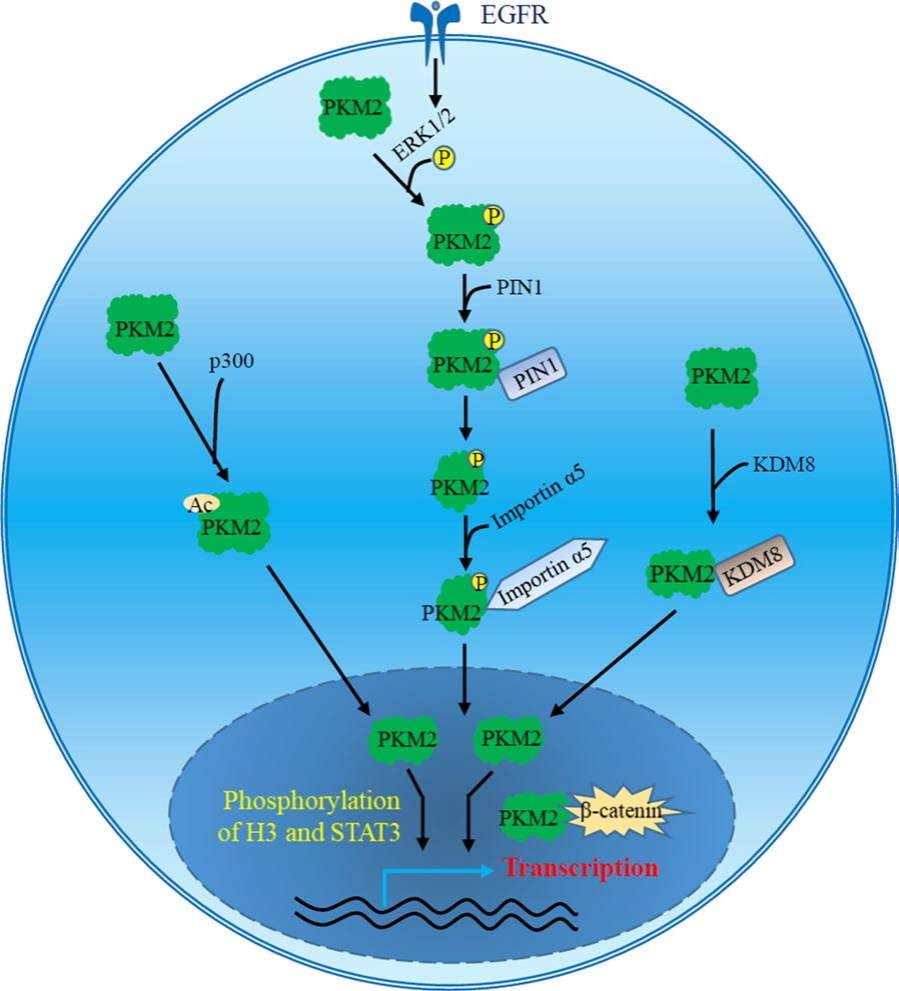
The proposed mechanism of PKM2 nuclear localization. PKM2 can be phosphorylated at Ser37 by ERK1/2 upon EGFR stimulation or acetylated at Lys433 by p300acetyltransferase. The conformation changes in response to post-translational modification favor the monomeric isoform of PKM2 and expose its nuclear localization signal, leading to nuclear localization. KDM8, a histone lysine demethylase, binds to PKM2, blocking PKM2 tetramer formation and promoting its nuclear translocation. Nuclear PKM2 serves as a protein kinase to phosphorylate histone H3 and transcription factor such as STAT3 or acts as a transcription coactivator to regulate the expression of glycolytic genes and other genes responsible for cell proliferation and tumor growth

Glycolysis takes place in the cytosol, where the involved enzymes activate their substrates and catalyze corresponding reactions. The PKM2-specific exon 10 encodes a nuclear localization signal (NLS), facilitating PKM2 import into the nucleus [23]. PKM2 was first reported in the nuclear extracts of tumors in 1988 [57], and many factors have been found to facilitate its translocation into the nucleus [58, 59]. Post-translational modification and conformation change are requirements for PKM2 nuclear translocation (Fig. 3). For example, EGFR signaling activates ERK1/2, which binds to PKM2 Ile429/Leu431 and phosphorylates PKM2 at Ser37 [23]. The PKM2 Ser37 phosphorylation recruits protein interacting with never in mitosis A-1 (PIN1) binding to PKM2. PIN1, a peptidyl-proline isomerase, specifically promotes cis-trans isomerization of the pSer37-Pro38 bond within PKM2. PIN1-dependent conformation change promotes the conversion of PKM2 tetramer to PKM2 monomer and exposes the PKM2 NLS for binding to importin α5, inducing PKM2 entrance to the nucleus [23, 60]. Importin α5 functions as an adaptor, facilitating the translocation of NLS-containing proteins through the nuclear membrane. Mutation of PKM2 Ser37 does not affect its pyruvate kinase activity but blocks its translocation into the nucleus as well as the EGF-induced Warburg effect and tumor development [23]. Phosphorylation of PKM2 at Thr454 has also been reported to facilitate its nuclear translocation and promote xenograft tumor growth [61].

Acetylation of PKM2 at Lys433 suppresses binding of FBP to PKM2 and the conversion of monomer or dimer to tetrameric form and enhances PKM2 nuclear import and protein kinase activity [62]. The shift of PKM2 from a metabolic enzyme in cytoplasm to a nuclear protein kinase potentiates cell proliferation and tumorigenesis. In contrast, SIRT6, a sirtuin family deacetylase, binds to and deacetylates nuclear PKM2 at Lys433, and SIRT6-mediated deacetylation facilitates export of nuclear PKM2 via exportin 4 transporter [63]. PKM2 deacetylation abolishes its nuclear protein kinase and transcription coactivator activities, leading to suppression of its nuclear oncogenic function and consequent tumor suppression and metastasis inhibition. KDM8, a histone lysine demethylase, binds directly to the interface area of PKM2 subunits, retarding PKM2 tetramer formation and promoting its nuclear translocation, where the KDM8/PKM2 complex acts as a coactivator of HIF-1α to induce the expression of glycolytic genes [64, 65]. The data suggest that PKM2 may enter the nucleus as a monomer. In addition, PIAS3-mediated SUMOylation of PKM2 enhances nuclear localization of PKM2, and overexpression of PIAS3 leads to the nuclear co-localization of PKM2 with PIAS3 [66].

The poly-ADP-ribose (PAR) signal and PAR polymerase play a role in base excision repair and are associated with tumorigenesis. Upon EGF stimulation, PKM2 enters the nucleus and binds to PAR [67]. The disruption of PKM2/PAR interaction via inhibition of PAR production or PKM2 mutation interferes with PKM2 nuclear retention and inhibits nuclear PKM2-mediated glycolysis and tumor growth, suggesting that PAR plays an important role in nuclear location and function of PKM2.

Nuclear PKM2 can serve as a transcriptional coactivator in cancer cells and contribute to the Warburg effect and tumorigenesis [23, 61, 62]. For example, nuclear PKM2 is a coactivator of β-catenin [23, 68]. Upon translocation into the nucleus, PKM2 is dephosphorylated at Ser37 through interaction with phosphorylated phosphatase Cdc25A at Tyr59, mediated by c-Src upon EGFR activation [68]. Dephosphorylation of PKM2 enhances PKM2-dependent β-catenin activity and c-Myc-mediated expression of the several glycolytic genes, facilitating the Warburg effect and tumorigenesis [68]. Deficiency of nuclear translocation in mutant PKM2 Ser37 inhibits the EGFR-mediated Warburg effect and tumor development in mice. PKM2 has been found to bind to nuclear HIF-1α, inducing the expression of *LDHA*, *PDK1*, and *SLC2A1* and promotes the metabolic switch from oxidative phosphorylation to glycolysis [69]. The prolyl hydroxylation of PKM2, but not PKM2 enzyme activity, is required for HIF-1-mediated transactivation in tumor cells [69]. Nuclear PKM2 has been identified as a coactivator of the transcription factor STAT5a, inducing cyclin D1 expression [70]. In addition, PKM2 has been found to bind to transcription factor Oct4, a critical regulator of stem cells, and modulate Oct4-mediated transcription, implicated in tumor cell growth and differentiation through the regulation of tumor cell stemness [21, 71].

## Protein kinase function of PKM2

PKM2-mediated gene transcription regulation may rely on its protein kinase activity that phosphorylates histones, transcription factors, and other signal molecules (Table 1). PKM2 acts as protein kinase with dual-specificity, phosphorylating both Ser/Thr and Tyr residues in protein substrates. Upon EGFR activation, PKM2 binds to histone H3 and acts as a protein kinase to phosphorylate H3 at Thr11 [30], an effect confirmed in yeast [72]. PKM2-dependent H3 phosphorylation facilitates the dissociation of histone deacetylase 3 from CCND1 and MYC promoters, resulting in subsequent acetylation of H3 at Lys9 and EGF-induced cyclin D1 and c-Myc transcription, which promotes cyclin D1-dependent cell cycle progression and tumorigenesis [30]. c-Myc expression upregulates the GLUT1, PKM2, and LDHA, and nuclear PKM2-mediated upregulation of these glycolytic proteins facilitates the Warburg effect and tumorigenesis [23]. This suggests a function of PKM2 as a protein kinase in its epigenetic control of gene expression and tumor development [23, 30].

**Table 1:** Select reported protein substrates of PKM2

Substrates	Amino acids	Functions	Reference
AKT1S1	Ser202, Ser203	mTORC1 signaling	He et al. [77]
Bcl2	Thr69	Apoptosis	Liang et al. [32]
Bub3	Tyr207	Cell cycle	Jiang et al. [28]
Histone H1			Ignacak and Stachurska [107]
Histone H3	Thr11	Gene transcription	Yang et al. [12]
Histone H2AX	Ser139	Genomic instability	Xia et al. [33]
MLC2	Tyr118	Cell cycle	Jiang et al. [29]
PAK2	Ser20, Ser141, Ser192, Ser197	Tumor invasion, metastasis	Cheng et al. [24]
SNAP-23	Ser95	Exosome release	Wei et al. [34]
STAT3	Tyr705, Thr454	Nuclear translocation, gene transcription	Gao et al. [20]; Yu et al. [61]
Interacting-partners when PKM2 serves as a transcription coactivator			
β-catenin			Yang et al. [19]; Yang et al. [23]; Liang et al. [68]
HIF-1α			Luo et al. [69]
Oct4			Lee et al. [71]; Morfouace et al. [21]
STAT5a			Park et al. [70]

AKT1S1, AKT1 substrate 1; Bcl-2, B-cell lymphoma 2; Bub3, spindle checkpoint protein; HIF-1α, hypoxia inducible factors-1 alpha; MLC2, myosin light chain 2; Oct4, octamer-binding transcription factor 4; PAK2, p21-activated kinase 2; SNAP-23, synaptosome-associated protein 23; STAT: signal transducers and activators of transcription

PKM2 can also directly phosphorylate transcription factor and regulate its transactivation activity [20]. Nuclear PKM2 has been demonstrated to phosphorylate STAT3 at Tyr705 using a phosphate group from PEP, subsequently activating transcription of MEK5 [20, 73] and HIF-1α [15]. The interaction of polypyrimidine tract-binding protein (PTBP1) and PKM2 facilitates phosphorylation of STAT3 Tyr705 and promotes oncogenesis in lymphoma [74]. PKM2-mediated STAT3 phosphorylation promotes progression of esophagus cancer via TGF-β1-mediated EMT [75]. A PKM2 mutant predominantly expressed as a dimer enhances cell proliferation, and the level of nuclear PKM2 is associated with cell proliferation, indicating an effect of PKM2 protein kinase activity on cell proliferation. Lipopolysaccharide facilitates PKM2 binding to the STAT3 promoter, subsequently promoting STAT3 transcription and its nuclear translocation and inducing pro-inflammatory cytokine secretion and cell proliferation in colorectal cancer [31]. Knockdown of STAT3 decreases PKM2-mediated inflammatory cytokine TNF-α and IL-1β expression that is dependent on PKM2 protein kinase activity but not pyruvate kinase activity. PKM2-mediated STAT3 nuclear translocation and the dimeric form of PKM2 showing protein kinase activity are also essential for colorectal cancer cell migration and adhesion [76]. The activation of STAT3 by nuclear PKM2 reduces the sensitivity of colorectal cancer cells to tyrosine kinase inhibitor of the EGFR pathway [42].

PKM2 can phosphorylate several key molecules associated with cell division and regulate cell cycle progression. In addition to being a histone kinase upregulating the expression of cyclin D1 promoting cell cycle progression described above [19, 30, 60], PKM2 binds to and phosphorylates Bub3 at Tyr207 during mitosis [28]. Bub3 is a spindle checkpoint protein, and its phosphorylation is essential for accurate chromosome segregation, proliferation of cancer cells, and active EGFR-induced brain tumorigenesis. Upon phosphorylation of PKM2 Thr45 by Aurora B, it binds to myosin light chain 2 (MLC2) in the contractile ring region of mitotic cells and, in turn, phosphorylates MLC2 at Tyr118 [29]. MLC2 phosphorylation induces the binding of Rho-associated protein kinase 2 (ROCK2) to MLC2 and the subsequent ROCK2-mediated phosphorylation of MLC2 at Ser15. PKM2-induced MLC2 phosphorylation is essential for cytokinesis and cell division as well as cancer cell proliferation and cancer development, highlighting the importance of PKM2 protein kinase function in oncogene-regulated cytokinesis and tumorigenesis [29].

PKM2 protein kinase activity can phosphorylate other signal molecules and be involved in multiple pathological processes in tumors. PKM2 phosphorylates mTORC1 inhibitor AKT1 substrate 1 (AKT1S1) at Ser202/203, and PKM2-induced AKT1S1 phosphorylation promotes PKM2 binding to 14-3-3, activating mTORC1 signaling and accelerating oncogenic growth in cancer cells [77]. PKM2 directly phosphorylates the p21-activated kinase 2 (PAK2) at four Ser residues and enhances PAK2 stability through PAK2 phosphorylation-induced HSP90 binding, facilitating the metastasis of pancreatic ductal adenocarcinoma [24]. Nuclear PKM2 binds to histone H2AX under conditions of DNA damage and phosphorylates H2AX at Ser139 [33]. Mutation of PKM2 kinase activity results in reduced cell proliferation and chromosome aberrations under conditions of DNA damage, suggesting that PKM2-mediated phosphorylation of H2AX increases genomic instability in cancer cells. PKM2 functions as a protein kinase to phosphorylate synaptosome-associated protein 23 (SNAP-23) at Ser95, which promotes exosomes release [34]. Mutation of SNAP-23 Ser95 impairs PKM2-induced tumor cell exosome release, suggesting a non-metabolic role of PKM2 in regulating tumor microenvironments via inducing exosome release. In addition, PKM2 can translocate to the outer mitochondrial membrane in response to oxidative stress, where it binds to and phosphorylates Bcl2 at Thr69 [32]. Phosphorylation of Bcl2 interferes with the binding of E3 ligase to Bcl2 and stabilizes Bcl2, inhibiting the apoptosis triggered by oxidative stress.

## Regulation of PKM2 activity

Activity of PKM2 can be modulated by covalent modification and allosteric regulation. As a protein kinase, PKM2 itself can be regulated by phosphorylation [78]. As mentioned, activated ERK1/2 phosphorylates PKM2 at Ser37, which promotes its nuclear translocation [23]. It has been found that PKM2 Tyr105 is frequently phosphorylated in human tumor response to fibroblast growth factor receptor signal [79]. Phosphorylation of PKM2 Tyr105 disrupts binding of FBP and prevents the formation of active, PKM2 tetramer. Tyr105 substitution has demonstrated that phosphorylation of PKM2 Tyr105 enhances the Warburg effect and tumor growth in xenografts in nude mice [79]. Phosphorylation of PKM2 Tyr105 by oncogenic kinases induces cancer stem-like cells and promotes tumorigenesis in breast cancer cells [80]. Centromere protein F, a microtubule binding protein involved in the chromosomal segregation during mitosis, rewires cancer metabolism through regulating PKM2 phosphorylation [81]. HSP90 induces phosphorylation of PKM2 at Thr328 via glycogen synthase kinase-3β (GSK-3β), which is critical to maintaining PKM2 stability and potentiating glycolysis and PKM2-mediated hepatocellular carcinoma growth [82]. AKT directly interacts and phosphorylates PKM2 at Ser202, which contributes to the nuclear import of PKM2 in response to insulin-like growth factor [70]. Nuclear PKM2 binds to STAT5a, facilitating the transcriptional activation of STAT5a target gene cyclin D1 and tumor growth [70]. Phosphorylated PKM2 easily dimerizes, promoting the release of exosomes [34]. Proviral insertion in murine lymphomas 2, a protein kinase acting as an oncogene, has been shown to phosphorylate PKM2 at Thr454, which facilitates the transcriptional co-activation of HIF-1α and β-catenin transcription factors to promote non-glycolytic nuclear function of PKM2 and tumor growth [83].

Several metabolic intermediates are allosteric effectors of PKM2. Besides FBP, a classic intermediate of the glycolytic pathway described above, succinyl-5-aminoimidazole-4-carboxamide-1-ribose-5’-phosphate (SAICAR) and serine can allosterically activate PKM2 [6, 84, 85]. SAICAR, an intermediate of de novo purine nucleotide biosynthesis, has been identified as a critical stimulator of PKM2. SAICAR is an abundant metabolite in proliferating cells, and the binding of SAICAR to PKM2 induces PKM2 protein kinase activity and phosphorylation of recombinant histone H3 [84]. SAICAR is essential for PKM2 nuclear localization, H3 phosphorylation, and c-Myc expression in tumor cells. SAICAR-mediated activation of PKM2 is critical for sustained proliferative signaling of cancer cells [84]. Proteomic analysis has demonstrated that many proteins associated with cell proliferation are phosphorylated by the PKM2-SAICAR complex [84]. ERK1/2 was identified as an important substrate. The PKM2-SAICAR complex phosphorylates and activates ERK1/2, which phosphorylates PKM2 and promotes SAICAR binding, forming a positive feedback regulation loop [84]. Introduction of a SAICAR-insensitive or a non-phosphorylatable PKM2 mutant inhibited the EGF-stimulated ERK activation and consequent cancer cell proliferation, suggesting SAICAR-induced PKM2 protein kinase activity is required for EGF-mediated cancer cell proliferation [84]. These findings suggest that PKM2-SAICAR protein kinase activity directly couples intracellular metabolic status with proliferation in cancer cells. A PKM2 variant (G415R) derived from a cancer patient was shown to bind to FBP, but cannot be activated by FBP, whereas it is activated by SAICAR [86]. Serine has been shown to be a natural ligand and allosteric effector of PKM2 [6]. PKM2 activity can be controlled through serine availability. Adequate serine activates PKM2 completely, enabling the maximum use of glucose via glycolytic pathway. Serine deprivation attenuates PKM2 activity, leading to the accumulation of glycolytic intermediates that feed into serine synthesis to facilitate cell proliferation [6, 85].

## PKM2 as a therapeutic target

Up-regulation of PKM2 is a hallmark of numerous tumor types, making it a potential therapeutic target [87]. Targeting PKM2 with some small molecules has been used in the preclinical studies to interfere with tumor growth. The strategy usually involves down-regulation of PKM2, blocking the nuclear translocation of PKM2 and promoting the tetrameric state of PKM2. For example, silencing PKM2 by specific siRNA increases tumor cell apoptosis and induces tumor regression in xenograft model [88, 89]. LY294002, a specific phosphatidylinositol-3-kinase inhibitor, inhibits gastric cancer cell proliferation and induces early apoptosis through the down-regulation of PKM2 [90]. Beta-elemene, a drug for complementary cancer therapy, inhibits breast cancer metastasis via blocking PKM2 dimerization and nuclear translocation [91]. As described above, targeting PKM2 also affects the sensitivity of cancer cells to chemotherapeutics. Knockdown of PKM2 increases 5-FU efficacy in colorectal cancer cells [92] and the sensitive of lung cancer xenograft to docetaxel [54]. PKM2 knockdown or drug inhibition makes resistant hepatocellular carcinoma re-sensitize to doxorubicin and cisplatin [93].

Small molecule PKM2 activators may also interfere with the metabolism of cancer cells for therapeutic purposes [94]. 2′-hydroxycinnamaldehyde, isolated from cinnamon, suppresses proliferation of prostate cancer cells through binding directly to PKM2 and blocking the phosphorylation of PKM2 at Tyr105. The decrease of the phosphorylation at Tyr105 inhibits protein kinase activity of PKM2 and increases its pyruvate kinase activity by facilitating the tetramer of PKM2, consequently reducing PKM2-mediated STAT3 phosphorylation and downstream target gene expression [95]. Therefore, PKM2 activators have therapeutic potential in the treatment of cancer [95, 96]. PKM2 activators induce tetramerization of two PKM2 dimers, causing PKM2 to function like PKM1 [96, 97]. Some activators that promote nuclear translocation of PKM2 and the conversion of PKM2 from dimer to tetramer or inhibitors that down-regulate the expression of PKM2 and inhibit the PKM2 activity could be promising anti-cancer drugs.

## Challenges against PKM2 protein kinase activity and its pro-tumorigenic effects

Although accumulating evidence demonstrates protein kinase activity of PKM2, the conclusion has recently been challenged [98–100]. A phosphoproteomic survey has identified 974 PKM2 substrates in the proteome of renal cancer [77], but the biochemical evidence of PKM2 protein kinase activity is still limited. When recombinant PKM2 and ^32^P labelled PEP were added to PKM2-deficient cell lysates, neither PKM2-dependent phosphorylation nor PKM2-dependent transfer of phosphate from ATP directly to protein were observed [98]. The results contradict a role of PKM2 as a protein kinase, although it may result from low levels of target substrate proteins and ^32^P-PEP in the reaction system [98]. In a previous study, PKM1 and PKM2 were quantified using mass spectrometry in many cancers and matched controls as well as cancer cell lines [100]. PKM2 was found to be a prominent isoform in all cancer and control samples, suggesting that PKM2 dominance was not a result of isoform switch during cancer formation [100].

The role of PKM2 in tumorigenesis has also been argued. A transgenic study revealed that PKM2 is not essential for BRCA1-deficiency-mediated breast cancer formation [101]. In contrast, PKM2 deficiency without disrupting PKM1 accelerated breast cancer formation in a mouse model of BRCA1 deficiency. PKM2 is not necessary for cancer maintenance and growth in vivo [102]. Mice lacking PKM2 are prone to spontaneous development of hepatocellular carcinoma due to inflammation and an imbalance in metabolism [103]. PKM2 absence did not affect c-Myc-mediated tumorigenesis in the liver, suggesting that PKM2 or PKM shift is not essential to facilitate c-Myc-induced tumorigenesis [99]. PKM1 has been revealed to be expressed in non-proliferating tumor cells but not in proliferating cells in PKM2 deficient tumors, suggesting that PKM2 is not required for cell proliferation and pyruvate kinase activity is necessary for non-proliferating tumor cells [101]. These data demonstrated that cells can modify PKM2 activity to meet the metabolic requirements of proliferating and non-proliferating cancer cells [101].

Discrepancies in results of PKM2 research may possibly be attributed to different experimental design or to cellular metabolic status. It has been reported that knock-down of either PKM isoforms in lung carcinoma cell lines H1299 and A549 resulted in different phenotypes, due to deficiency in AMP-activated protein kinase signaling in A549 cells [104]. Differential regulation of PKM2, inactivation or activation, by A-Raf in primary mouse fibroblasts or immortal NIH3T3 fibroblasts is reported dependent on metabolic flux of glutamine and serine [105].

## Conclusions

Emerging evidence suggests that some metabolic enzymes that phosphorylate metabolic intermediates can also act as protein kinases to phosphorylate various protein substrates in multiple biological processes [106]. PKM2 is highly expressed in many types of tumors and dominant expression of the low-activity dimeric isoform of PKM2 is considered critical for aerobic glycolysis in tumor cells and tumor growth [104]. PKM2 has been proposed to exert dual roles in tumor cells: metabolic functions serving as a pyruvate kinase to control cancer cell metabolism and non-metabolic function acting as a protein kinase to regulate gene expression required for cell proliferation [19]. PKM2 dimer has been suggested to serve as a protein kinase, while the tetramer acts as a pyruvate kinase [20].

As described above, the effects of PKM2 in tumorigenesis remain controversial. Although the moonlighting phenomenon of a metabolic enzyme is not rare in biochemistry, contradictory data on PKM2 protein kinase activity need to be clarified by providing further solid biochemical evidence and elucidation of function *in vivo*. With respect to this, several important issues are still unresolved. First, the elucidating the evolutionary origin of PKM2 protein kinase function may help to characterize the complex PKM2 functions. Secondly, key evidence of the PKM2 protein kinase activity contribution to tumorigenesis has been produced by research on brain tumors [12, 19, 23, 30]. Brain mainly obtains energy from glucose catabolism, and glycolysis is vigorous in brain tissue. Current data does not confirm tumor specificity of PKM2 activity. Finally, the PKM2-mediated balance of metabolism and proliferation is required for tumorigenesis [19], but underlying mechanisms are not well understood. Although PKM2 may undergo a function shift according to cellular metabolic status [84], this needs further investigation. Furthermore, whether there is crosstalk between PKM1 and PKM2 is also an open question. As far as we know, there seems to be no direct evidence that PKM1 regulates PKM2 function or vice versa. Fully understanding PKM2 function in cancers will help characterize metabolic reprogramming of tumors, providing targets for tumor therapy.

## Data Availability

Not applicable.
